# Red cabbage extract-mediated colorimetric sensor for swift, sensitive and economic detection of urease-positive bacteria by naked eye and Smartphone platform

**DOI:** 10.1038/s41598-023-28604-1

**Published:** 2023-02-04

**Authors:** Cagla Celik, Naim Yagiz Demir, Memed Duman, Nilay Ildiz, Ismail Ocsoy

**Affiliations:** 1 Department of Analytical Chemistry, Faculty of Pharmacy, Erciyes University, Kayseri, 38039 Turkey; 2 Pharmacy Services Program, Vocational School of Health Services, Hitit University, Corum, 19000 Turkey; 3 Nanotechnology and Nanomedicine Division, Institute of Science, Hacettepe University, Ankara, 06800 Turkey; 4 Department of Pharmaceutical Microbiology, Faculty of Pharmacy, Erciyes University, 38039 Kayseri, Turkey

**Keywords:** Biochemistry, Biological techniques, Biotechnology, Microbiology, Medical research, Chemistry

## Abstract

The bacterial pathogens have caused various serious infectious diseases in the human body, and even some threats to human life by leading to deaths. *Enterobacteriaceae* species especially urease positive ones, *Proteus mirabilis* (*P. mirabilis*) and *Klebsiella pneumoniae* (*K. pneumoniae*), show resistance to antibiotics and cause respiratory and urinary tract infections. We have developed natural indicator-incorporated colorimetric urease tests with a naked eye and smartphone readout to rapidly, sensitively and economically detect *P. mirabilis* and *K. pneumoniae*. We utilized anthocyanin found as a predominant component in red cabbage (*Brassica oleracea*) extract as a natural pH indicator instead of toxic and synthetic indicators. As a mechanistic explanation for the detection of *P. mirabilis* and *K. pneumoniae*, urease enzymes secreted from the *P. mirabilis* and *K. pneumoniae* hydrolyze urea to produce ammonia (NH_3_), which increases the pH value of the reaction environment and leads to deprotonation from anthocyanins. The changes in the molecular structure and electronic structure of anthocyanins are responsible for revealing many different colors. We demonstrated how some reaction parameters including the concentration of the bacteria (colony-forming unit, CFU), the concentration of anthocyanin in the tests, initial color and pH values (pHs) of the tests influence their detection performance. We further developed a 3D-printed smartphone platform with smartphone based digital image processing software to improve the detection limit and shorten the detection time. We claim that natural indicator-incorporated rapid urease tests providing colorimetric readout evaluated by the human eye and smartphone imaging processing has great potential in practical use and they can be implemented in clinics.

## Introduction

Although *Proteus mirabilis* (*P. mirabilis*) and *Klebsiella pneumoniae (K. pneumoniae)* strains are harmlessly found in human fecal flora and whole gastrointestinal tract^1^, they separately or together colonize in respiratory and urinary tracts and cause severe single or co-infections by forming biofilm^2–4^. These urease positive and gram-negative bacteria have developed resistance against extended-spectrum antibiotics, which increases anxiety in the medical community. In addition to these life-threatening infections caused by these pathogens, they indirectly induce the formation of bladder and kidney stones. Simply, the urease enzymes released from the pathogens hydrolyze the urea to form ammonia (NH_3_), which forms the various sized crystals consisting of insoluble polyvalent anions and cations in alkaline urine^5–8^.

In addition to these issues aforementioned, today, immediate prescription of broad-spectrum antibiotics and wrong treatment until detection of these pathogens are also other crucial problems, that not only adversely affect patients’ health but also induce the emergence of increased resistance. To address all these issues, there is a high demand to develop rapid and sensitive tests for detection of the pathogens. Although there are several genotypic methods currently used in clinics, the needs for expensive devices and/or specialists are major disadvantages for these methods^9–11^.

Herein, we developed natural indicator-based urease tests, for the first time, to rapidly, sensitively and colorimetric detection of the growth of *P. mirabilis* and *K. pneumoniae* strains with a naked eye and smartphone based digital imaging processing system. The indicator, anthocyanin molecules, is especially based on the structure of cyanidin 3-O-diglucoside-5-O-glucoside, obtained from red cabbage extract (RCE). The anthocyanins are biocompatible and give response with various colors to very small pH changes. Taking advantage of these properties of anthocyanin, it has been used in bacterial diagnostic tests^12^. Since the anthocyanins have a wide pH-dependent color scale, the initial color of the test can be designed.

While the observation of changes in the test solution color with aid of the naked eye provided qualitative analysis in the detection of bacterial growth or presence, semi-quantitative results were achieved by smartphone based digital imaging systems including Delta-E (ΔE) and RGB (Red Green Blue) analysis. We promisingly offer that both readout systems can be considered as relatively cost-effective and portable tools and can be implemented in point-of-care (POC) testing and in clinics for the detection of various microorganisms.

## Materials and methods

### Materials

Triptic soy agar (Merck, Germany), Columbia agar base (BD, Germany), skimmed milk medium (Difco, USA), Sodium dihydrogen phosphate monohydrate (NaH_2_PO_4_.H_2_O, Merck, Germany), Sodium phosphate dibasic anhydrous (Na_2_HPO_4_, Merck, Germany), Sodium azide (NaN_3_, Merck, Germany), and urea (Merck, Germany) were purchased from the company stated. Microorganism: *Escherichia coli ATCC 25922 (E. coli), K. pneumoniae ATCC 700603* and *P. mirabilis ATCC 12453* were obtained from Erciyes University, Faculty of Pharmacy, Pharmaceutical Microbiology research laboratory ATCC culture collection. *P. mirabilis* and *K. pneumoniae* were stored in skim milk medium at -20 °C and regenerated prior to the experiments. The optical density was determined by spectrophotometer (AzureAo, Azure Biosystems, Inc.).

### Preparation of red cabbage extract and natural indicator-incorporated test

The commercialized red cabbage was purchased from the local Mimar sinan market (38°43′38.3"N 35°31′30.6"E) in the province of Kayseri in Turkey. The red cabbage used in the research are permitted and legal for trade, commercialization in Turkey. Therefore, specific permission was not needed from the Local Authority. Red cabbage leaves were cut into small pieces by passing through a shredder. The 100 gram (g) of material was placed in a 500 mL beaker containing 100 mL of distilled water. The mixture was exposed to the extraction process by boiling it for 30 minutes (min). The resulting red cabbage leaves extract was filtered and stored at -20 °C for further use.

To prepare the anthocyanin-based rapid urease test, firstly 0,01 M PBS was sterilized in an autoclave at 121 °C for 15 min. Then, the test solution was prepared by mixing 100 mL of 0,01 M sterilized PBS, 2 g of urea, 20 mg of NaN_3_ and 20% w/w of RCE. Finally, the resulting test solution was filtered through a membrane filter with a pore size of 0.45 µm prior to use for bacteria detection.

### Bacterial strains and culture conditions

*K. pneumoniae* and *P. mirabilis* strains were grown at 37 °C in tryptic soy agar and Columbia agar base containing 5% sterile sheep blood. *K. pneumoniae* and *P. mirabilis* strains were suspended in 3 mL saline at different turbidities^13,14^. *E. coli* strain was cultured based on the reported protocol^15^.

### Digital image processing

For digital image processing, tubes containing anthocyanin test solution were placed on a white background and the color changes were captured with a smartphone camera (Apple iPhone 7 plus) and all photos were saved in JPEG format. Anthocyanin test solution images were analyzed by ImageJ software (National Institutes of Health) to quantitatively support the visual colorimetric results. RGB (Red Green Blue) analysis mean values and Euclidean distance were calculated using ImageJ software. With this software, all pixels in the captured images were separated into red, green and blue components, and the average values of the R, G or B channels were calculated^16,17^. The other analytical method used in quantitative analysis is based on measuring the color differences between two images by applying the Euclidean distance formula Delta-E (ΔE) obtained from the CIE 1976 Lab color difference formula^17^.

1 $$\Delta E = \left[ {\left( {\Delta L} \right)^{2} + \left( {\Delta a} \right)^{2} + \left( {\Delta b} \right)^{2} } \right]^{1/2}$$

L, a and b in the formula represent the dimensions of the CIE Lab color space, where the L axis represents the aperture in the range of black (0) to white (100). The axis ranges from red (+ a) to green (− a), and the b axis describes yellow (+ b) to blue (− b). Based on this information, ΔE calculates a lower score for similar images, since the distance that expresses the rank difference between similar images is smaller than the distance between different images^18,19^. To calculate ΔE; ΔL, Δa and Δb values were calculated by subtracting the L, a and b channels of the test result image and reference images, respectively. In the next step, ΔE was calculated for each pixel using Eq. 1. The ΔE value is accepted as an indicator by which the human eye can distinguish two colors under normal conditions^20^. Therefore, larger differences in ΔE values enable a more accurate conclusion of bacterial presence.

### Development of a smartphone application

A custom-built smartphone application with an image processing function was developed in Kotlin using Android Studio IDE. The application has the ability to run from Android 6 (API 23) to Android 10 (API 29). The mobile app must have an ethernet connection to allow access to the camera and files on the device. The main menu consists of a “Urease Test” button to start the menu. This interface contains guiding information on how to insert the test tubes and how to capture the image for analysis. After photographing the sample tube and reference tube, the Red Green Blue (RGB) values of each test image are calculated. The Euclidean distance formula works using these values. After analysis, all RGB values, Euclidean distance result and the final result of the test can be seen at the bottom of the screen. Euclidean distance (ED) formula (Eq. 2) is given below^21^.

2 $${\text{ED}}^{2} = (R_{2} - R_{1} )^{2} + (G_{2} - G_{1} )^{2} + (B_{2} - B_{1} )^{2}$$

For values of 25 and above, positive text appears on the screen, thus confirming the presence of urease-positive bacteria. To obtain reliable and highly accurate results, (i) both sides of the screen should be evenly lit when taking the picture, and (ii) the camera should be kept in the same position whenever possible to avoid focusing on the background or other things. This may not always be possible. For this reason, a smartphone platform has been developed to standardize the distance of the camera to the tubes and to obtain an equal lighting environment. Reliable results can be obtained thanks to the platform that ensures that all environmental factors are equal when photographing each sample.

### Fabrication of 3D-printed smartphone platform

The smartphone platform was designed via computer-assisted design (CAD) software SolidWorks according to Vestel Venus Z20. The Venus Z20 has 16-megapixel and 5-megapixel rear cameras. The smartphone platform was 3D-printed with Ultimaker S3 by using black tough poly(lactic acid) (PLA) as body material and white poly(vinyl alcohol) (PVA) as support. In order to prevent light leakage, the filament was chosen as black, and the infill was 20%. After the 3D printing, which consists of three parts, the bottom and the top of the platform and vial holder, it was left in water overnight to purify it from the support material by dissolving PVA. The white Light Emitting Diode (LED) strip was placed opposite the window of the vial holder. The power cable was routed through the hole in the base of the bottom part and connected to an on–off switch and then the 12 V adapter. Then, the upper part is fixed to the lower part with 3M 468MP double-sided tape. As a final product, the smartphone platform has a dimension of 150 mm × 120 mm × 160 mm and a weight of 300 g. The vial holder has two rings for vials, one for control and the other for sample. The vials were photographed by placing the phone in the 140 mm × 60 mm recess on the front of the platform.

### Statistical analysis

Analysis of the data was carried out using the SPSS 20.0 program. Data were expressed as the mean ± standard error of mean (SEM).

## Results and discussion

**Scheme 1:**
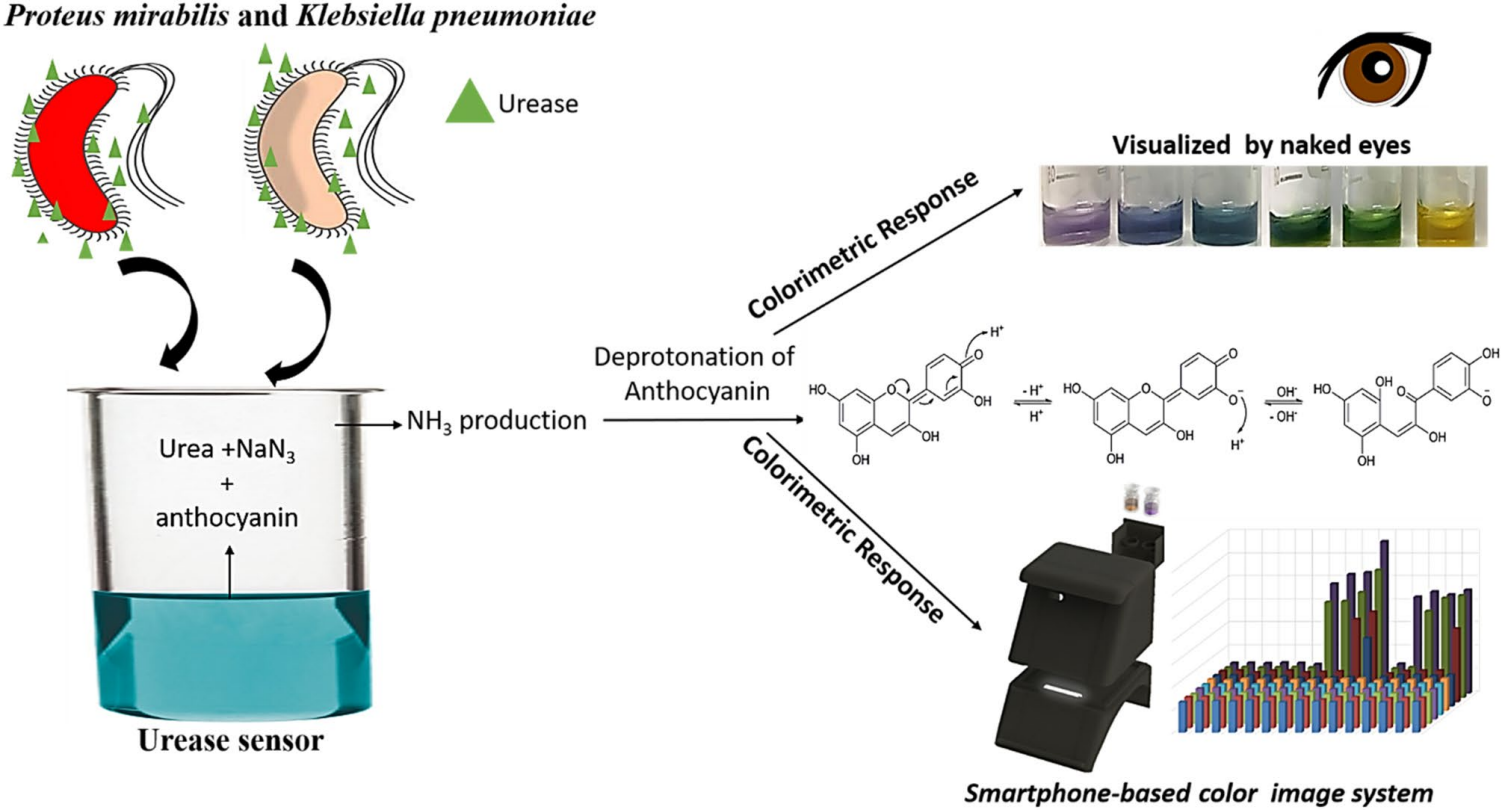
Illustration of a novel and colorimetric urease test based detection of *P. mirabilis* and *K. pneumoniae* with a naked eye and smartphone readouts.

The urease test is consisting of three major components including (i) urea used as a main substrate for production of NH_3_, (ii) anthocyanin acting as an indicator benefiting from its pH dependent color change property and (iii) NaN_3_ functioning as a stabilizer for prevention of self-hydrolysis of urea. The anthocyanin allows us to design test color based on starting pH values of the test environment. pH dependent color change relies on deprotonation from hydroxyl groups of anthocyanin. The working principle of this urease test and readout systems were illustrated and well documented in the Scheme 1. Both bacteria, *K. pneumoniae* and *P. mirabilis*, known as urease positive bacteria release urease catalyzing the hydrolysis of urea to NH_3_ molecules to make the reaction environment alkaline. Then, anthocyanin molecules are deprotonated through their hydroxyl groups and the color changes of the urease tests were systematically observed with a naked eye and smartphone-integrated imaging system.

We firstly prepared three urease test solutions as termed test 1 (at pH 6.5 with light purple color), test 2 (at pH 8 with blue color), and test 3 (at pH 11 with green color) with similar contents (10% w/w RCE:PBS (1:4), 2 µg/mL colistin sulfate, urea and NaN_3_). We demonstrated how initial color or pH of colorimetric urease tests influence their detection performance in terms of rapidity and sensitivity. The color changes in these tests were observed with a naked eye and digital image processing software (ΔE and RGB analysis) in colorimetric detection of *E. coli, K. pneumoniae* and *P. mirabilis* strains. In terms of experiment, 50 µl of 1.5 McFarland bacterial suspensions (*E. coli, K. pneumoniae* and *P. mirabilis*) were separately added into test 1, test 2 and test 3, then all of which was incubated for 180 min.

**Figure 1:**
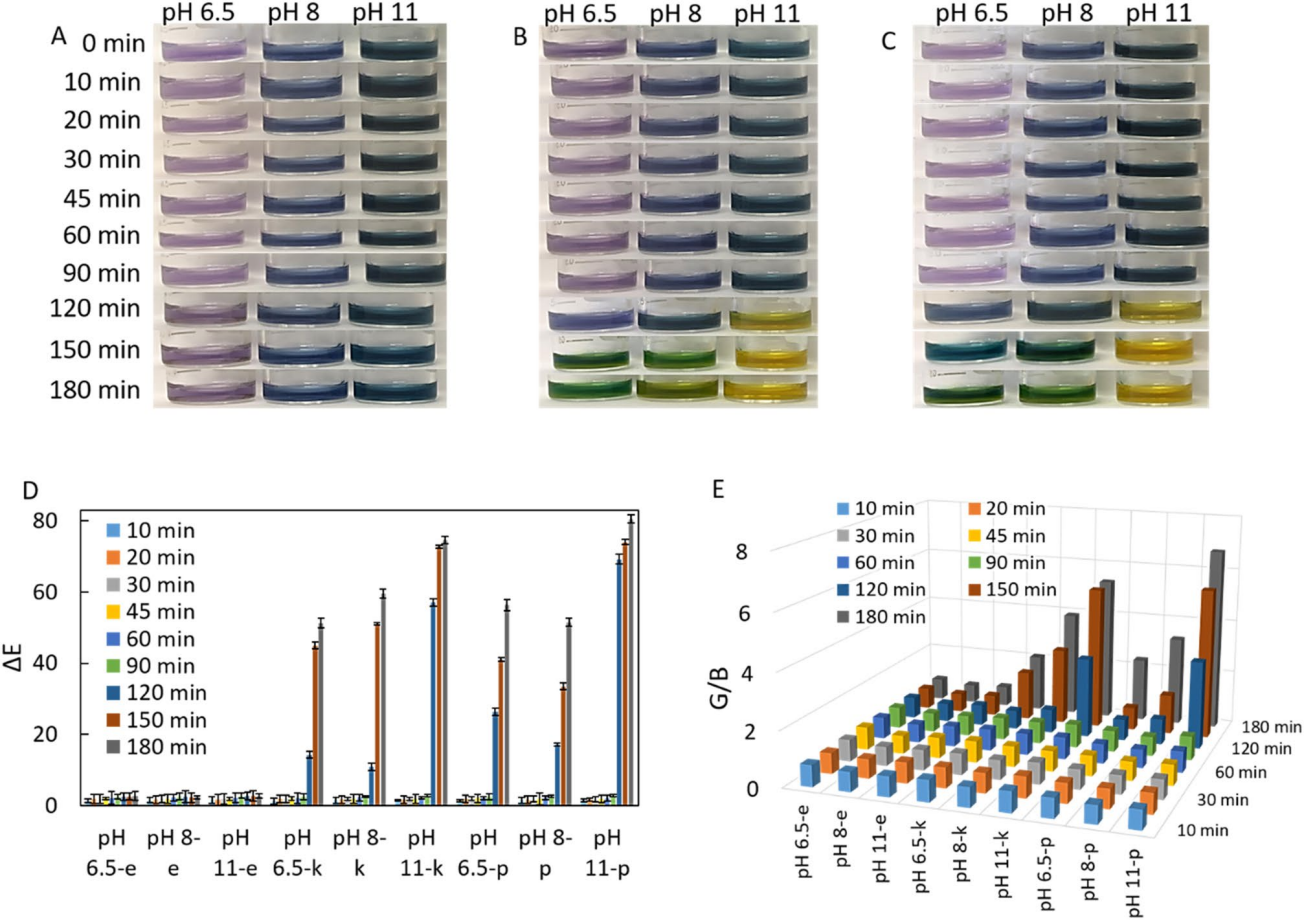
Colorimetric readouts of test 1, test 2 and test 3 with a naked eye for detection of 1.5 McFarland bacterial suspension of (**A**) *E. coli,* (**B**) *K. pneumoniae* and (**C**) *P. mirabilis*. (**D**) with ΔE values and E) G/B values. Not: pH 6.5-e, pH 8-e and pH 11-e represent presence of *E. coli* in the tests adjusted to those pH values; pH 6.5-k, pH 8-k and pH 11-k represent presence of*. K. pneumoniae* in the tests adjusted to those pH values; pH 6.5-p, pH 8-p and pH 11-p represent presence of *P. mirabilis* in the tests adjusted to those pH values. The error bars demonstrate one standard deviation (SD) obtained from three independent measurements (n = 3).

Figure 1A shows that *E. coli* was not grown owing to antibiotic (colistin sulfate) in all tests, then no change was seen in the initial color of each test owing to inhibition of *E. coli* in the presence of the antibiotic. *E. coli* was used as control bacteria because it is a urease-negative bacteria that often causes urinary tract infections. *E. coli* is a urease negative bacteria and does not secrete urease enzyme. However, in the experiments where we did not add antibiotics, we observed that the color of the tests with *E. coli* shifted to pink. Pink color indicates that the medium is acidic. This situation shows that *E. coli* releases acidic volatile components to the environment due to the continuation of its vital activities. As can be seen in the previous studies of the authors of this article, if bacteria continue to reproduce and continue their vital activities, they release acidic volatile compounds to the environment^22,23^. Knowing this situation, we inhibited *E. coli* and kept the color of the test solution stable. Urease enzyme activities of *Klebsiella pneumoniae* and *Proteus mirabilis* strains continue in the presence of antibiotics. When the same protocol was followed used for *P. mirabilis, K. pneumoniae* changed both the initial color and pH of each test in the incubations of 120. min, 150. min and 180. min (Fig. 1B,C). For instance, initial light purple color of test 1 (pH 6.5), blue color of test 2 (pH 8), and green color of test 3 (pH 11) was turned to bluish (pH 7.5), greenish (pH 8.5) and yellow (pH 12.5) at 120. min incubation, to green (pH 10), green (pH 11) and yellow (pH 13) at 150. min incubation and to green (pH 11), yellowish (pH 12) and yellow (pH 13) at 180. min incubation, respectively. The color and pH changes in all tests as observed for *K. pneumoniae* were almost similar in the detection of *P. mirabilis* (Fig. 1C). The ΔE and RGB analysis of each test was well consistent with color changes evaluated by a naked eye (Fig. 1D,E). *E. coli* caused no increase in both ΔE and G/B values for all tests due to the initial color of each test were unchanged. The differences between ΔE and G/B values were increased starting from the incubation of 120. min in detection of *K. pneumoniae* and *P. mirabilis* strains.

**Figure 2:**
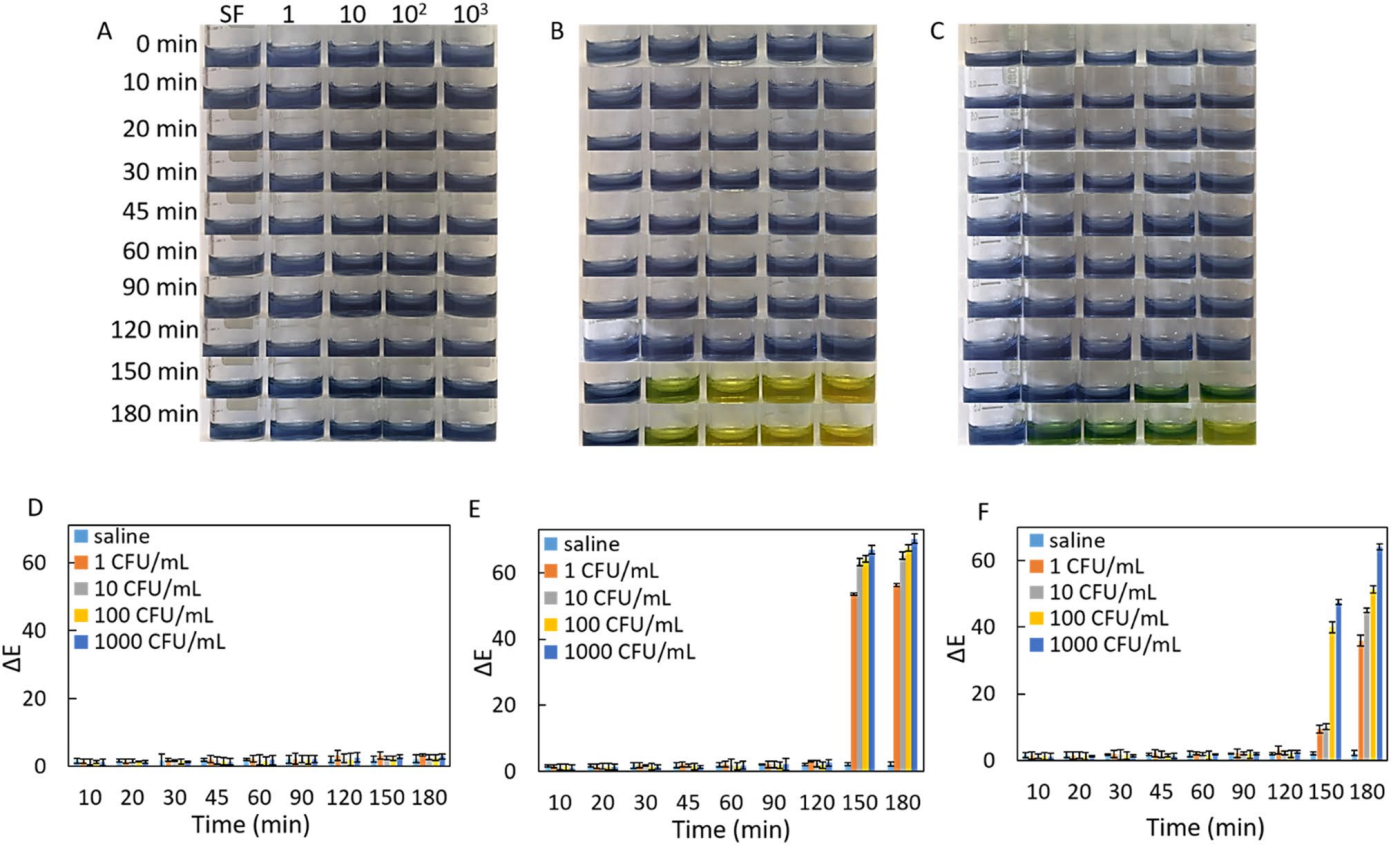
Colorimetric readouts of test 2 with a naked eye for (**A**) *E. coli,* (**B**) *K. pneumoniae* and (**C**) *P. mirabilis*. With ΔE values for (**D**) *E. coli,* (**E**) *K. pneumoniae* and (**F**) *P. mirabilis*. The error bars demonstrate one standard deviation (SD) obtained from three independent measurements (n = 3).

We studied the performance of test 2 (prepared at pH 8) and test 3 (prepared at pH 11) in the detection of *K. pneumoniae* and *P. mirabilis* as a function of the bacterial concentration. To 100 µl of test 2, 50 µl of each bacterial suspension (*E. coli, K. pneumoniae* and *P. mirabilis*) with a series of CFU/mL (1, 10, 10^2^ and 10^3^) was separately added. Each test 2 was incubated for 180 min, the potential colorimetric readout was evaluated with a naked eye (Fig. 2A–C) and smartphone based ΔE analysis (Fig. 2D–F) in certain time intervals. There is no color change was observed in the initial blue color of only saline (SF) included in test 2 during the incubation from 0. min to180. min at room temperature (RT:25 °C), which is an indication of the stability of test 2 as shown in the first column of Fig. 2A–C. The growth of *E. coli* strains prepared with various concentrations was fully inhibited in test 2 owing to the use of antibiotic, then initial blue color of test 2 remained constant at all incubation times as witnessed by a human eye in Fig. 2A.

The *K. pneumoniae and P. mirabilis* suspensions were separately added to each test 2 by following the same protocol for *E. coli.* Figure 2B demonstrated that the 1 CFU/mL *K. pneumoniae* was not able to change the color of test 2 even at 180. min incubation owing to not enough urease release. In addition to that, 10, 100 and 1000 CFU/mL *K. pneumoniae* changed the color of test 2 from blue to yellowish and yellow at 150. min of incubation, respectively (Fig. 2B). We interpret that amount of urease release is directly related to the number of CFU/mL of *K. pneumoniae*, which leads to hydrolysis of more urea and eventually production of more NH_3_ molecules. The starting pH of test 2 (pH 8) rises to till above pH 11 based upon NH_3_ concentration, and then the number of deprotonated hydroxyl groups increases. Figure 2C revealed that although *P. mirabilis* also urease positive bacteria, 1 and 10 CFU/mL *P. mirabilis* did not change the color of test 2 till 150. min incubation, color change from blue to green was seen at 180. min incubation as the earliest time. While 100 CFU/mL *P. mirabilis* strains resulted in the green color at both 150. and 180. min of incubation times, 1000 CFU/mL *P. mirabilis* changed the color of test 2 to green and yellowish at 150. and 180. min of incubation times, respectively. Both *K. pneumoniae* and *P. mirabilis* strains with all concentrations (CFU/mL) produced much urease enzyme at the end of the 12 h incubation, then the reaction environment was brought to quite an alkaline condition (pH > 12) and starting color of the test 2 (blue color at pH 8) was turned to bright yellow as shown in the supporting materials (Figure S1).

Delta-E (ΔE) analysis was systematically performed using a lab-made 3D printed smartphone platform to quantitatively support the colorimetric readouts and all are quite consistent with naked eye analysis. For instance, the *E. coli* strain did not change the color of test 2 in any concentrations (CFU/mL) and at any incubation times, so no differences between ΔE values were calculated as seen in Fig. 2D. In contrast to that, based on color changes caused by *K. pneumoniae* strains at all concentrations (CFU/mL) were observed at 150. min and 180. min of incubation, remarkably high ΔE values were produced at corresponding incubation times (Fig. 2E). Although a remarkable increase in ΔE values was recorded in the presence of *P. mirabilis* strains, the kind of higher increase in ΔE value was observed in the presence of *K. pneumoniae* compared to *P. mirabilis* due to much production of urease by *K. pneumoniae* strains (Fig. 2F). Although no clear color change was observed in the initial blue color of test 2 with a human eye till 150. min of incubation when 1 and 10 CFU/mL *P. mirabilis* were used, a significant increase in ΔE values was observed with digital imaging processing analysis. This case indicates that the color imaging processing system detects the presence of the corresponding pathogen when even the human eye fails. It is worth to mention that our colorimetric results uncovered much urease production performance of *K. pneumoniae* strains compared to *P. mirabilis* strains^24^.

**Figure 3:**
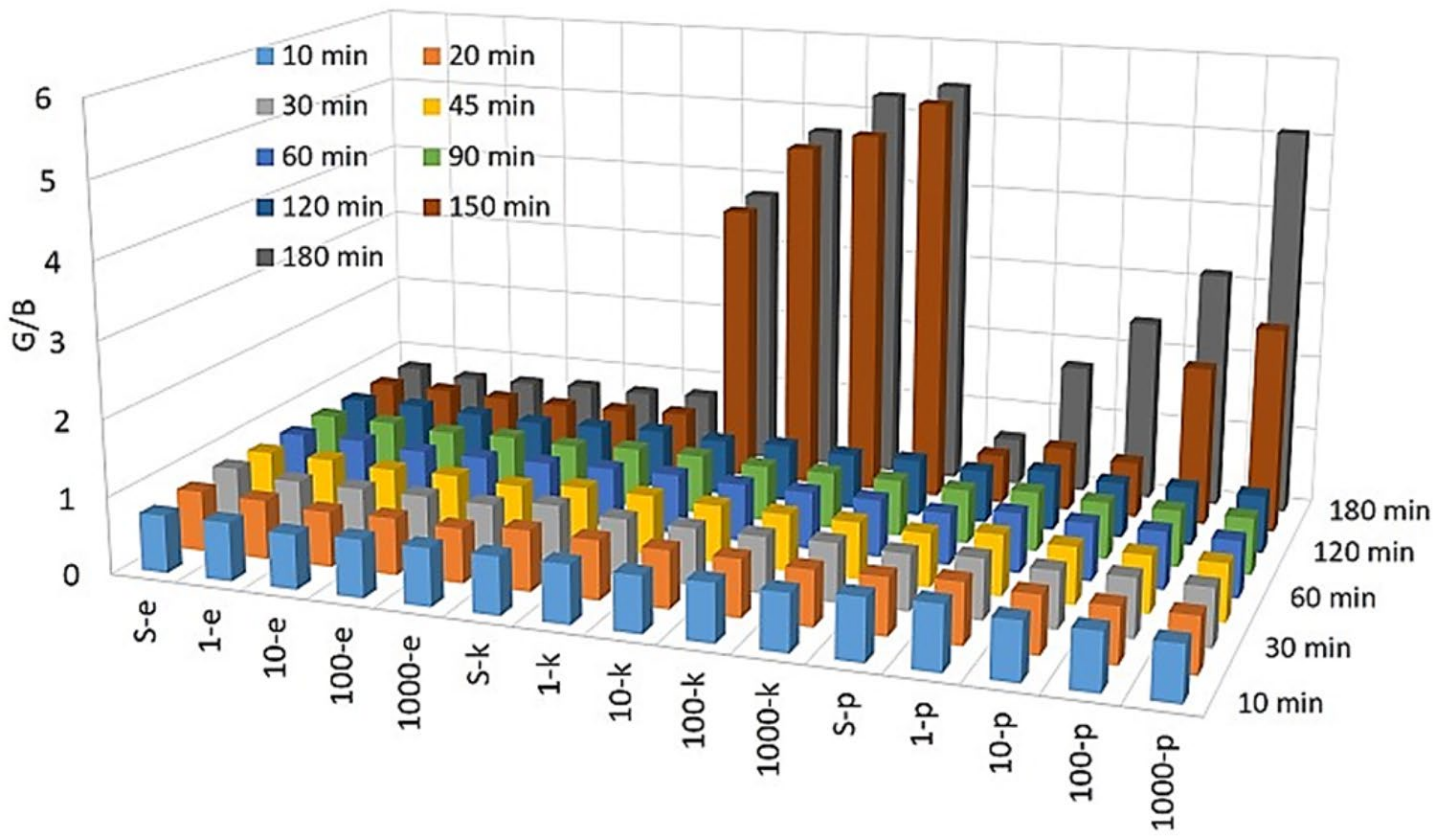
Colorimetric readouts of test 2 with RGB analysis for detection of *E. coli, K. pneumoniae* and *P. mirabilis*.

As a supporting study for ΔE, we employed RGB analysis to determine the level of the color difference caused by *E. coli, K. pneumoniae* and *P. mirabilis* strains. Figure 3 shows that *E. coli* strains did not result in any changes even in the level of the color of test 2 in any concentration and incubation time. However, *K. pneumoniae* and *P. mirabilis* induced the distinct color change at 150. min and 180. min incubations with certain bacterial concentration, which was calculated in the RGB analysis based on the increase in G/B values. A much increase in G/B values was observed in the presence of *K. pneumoniae* compared to *P. mirabilis* strains dependent upon the amount of released urease.

**Figure 4:**
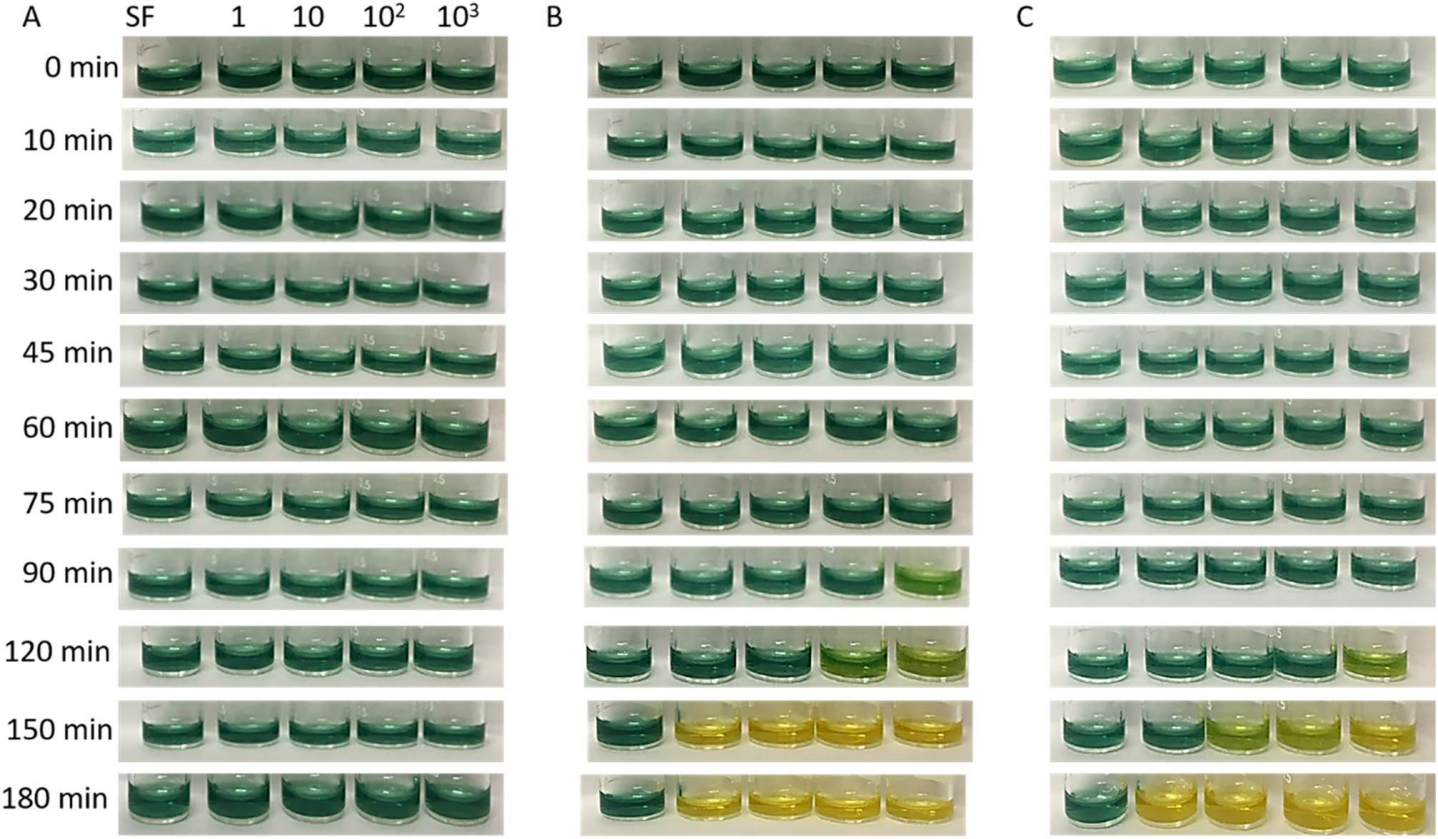
Colorimetric readouts of test 3 with a naked eye for (**A**) *E. coli,* (**B**) *K. pneumoniae* and (**C**) *P. mirabilis*.

We further studied the effect of bacteria concentrations (CFU/mL) on the performance of test 3. The color of test 3 without bacteria exhibited no change in its original green color, regardless of incubation times, which indicates its great stability (SF column in Fig. 4A). As expected, the inhibition of *E. coli* growth did not allow changing the pH of the reaction environment of test 3, then the initial green color of test 3 remained unchanged as visualized with a human eye in Fig. 4A. In contrast to test 2, the visible color change was rapidly seen in 90. min incubation in test 3 from green (pH 11) to yellowish (pH 12) in the presence of the 1000 CFU/mL *K. pneumoniae* (Fig. 4B). While the yellowish color of test 3 was also observed with 100 CFU/mL *K. pneumoniae* at the incubation of 120. min, at all concentrations of *K. pneumoniae*, the pH of the reaction medium raised above 12 and the color of test 3 turned bright yellow. The reaching to yellow color means that all hydroxyl groups of anthocyanin molecule were deprotonated, and even aromatic ring was opened via hydrolysis at over pH 12. Figure 4C one more time demonstrated that *P. mirabilis* releases much less and slowly urease compared to *K. pneumoniae*, then this case may affect the detection performance of test 3. For instance, the first color change in test 3 was witnessed with the naked eye in presence of 1000 CFU/mL *P. mirabilis* at the incubation of 120. min.

Although yellow color of test 3 was captured with all concentration of *K. pneumoniae* including 1000, 100, 10 and even 1 CFU/mL *K. pneumoniae* at the incubation of 150. min, but only 1000 CFU/mL *P. mirabilis* was able to change the initial green color of test 3 to yellow, both 100 and 10 CFU/mL *P. mirabilis* resulted in yellowish color, and unfortunately no color change was seen with use of 1 CFU/mL *P. mirabilis* at the same incubation time. Interestingly, the color of test 3 was turned in the presence of all concentration of CFU/mL *P. mirabilis* at 180. min of incubation.

**Figure 5:**
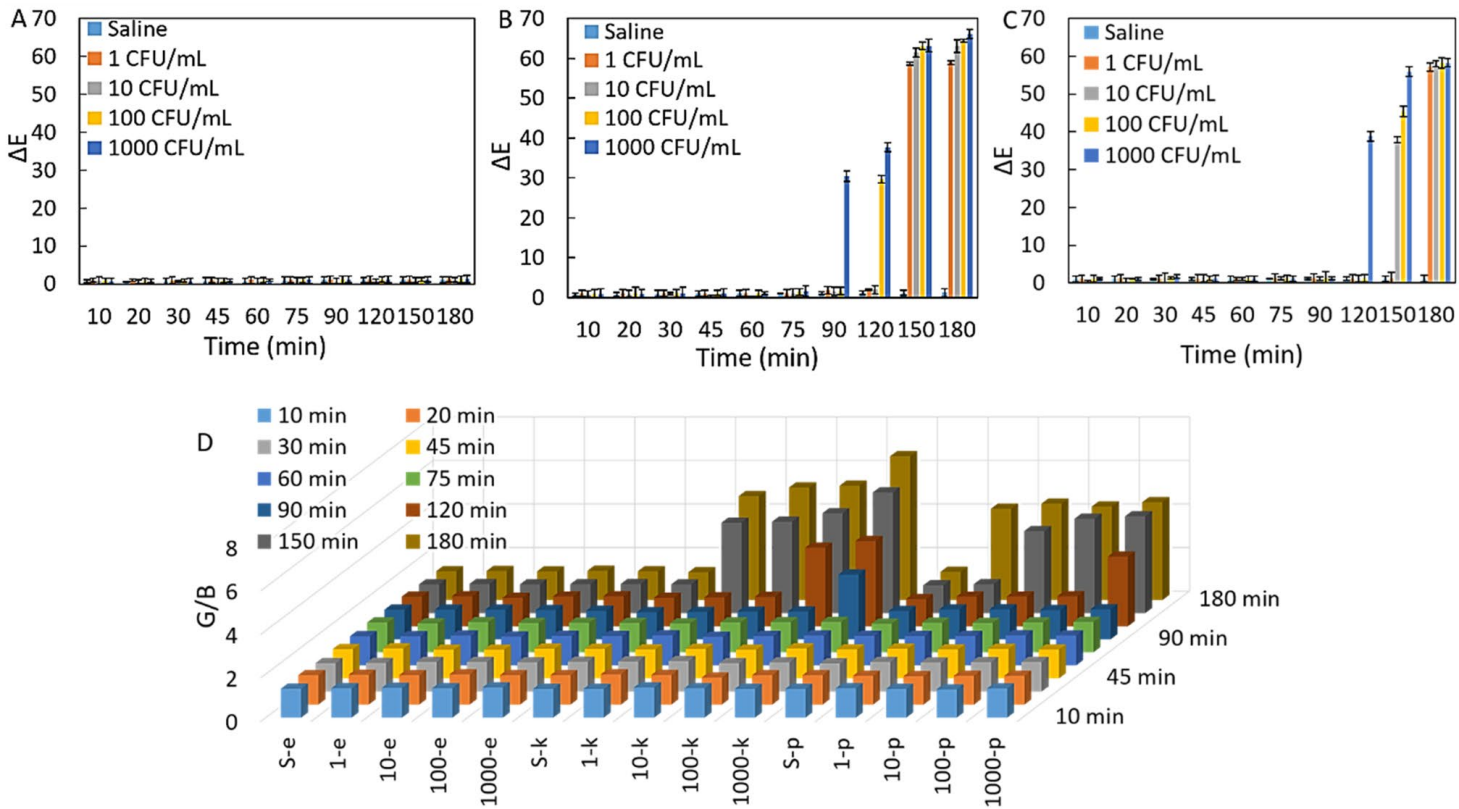
Colorimetric readouts of test 3 with ΔE values (**A**) *E. coli,* (**B**) *K. pneumoniae* and (**C**) *P. mirabilis*. (**D**) G/B values for all pathogens. The error bars demonstrate one standard deviation (SD) obtained from three independent measurements (n = 3).

As an additional study, colorimetric readouts of test 3 were also analyzed with ΔE and RGB calculation. *E. coli,* which did not cause a visible color change in test 3, did not make a difference in ΔE and G/B values either as shown in Fig. 5A,D, respectively comparing with Fig. 5B,C. The increase in both ΔE and G/B values in detection of *K. pneumoniae* and *P. mirabilis* strains were well consistent with naked eye evaluation of color of test 3 as presented in Fig. 4A–C. The first color differences in ΔE and G/B values are clearly shown at after 90. min incubation of 1000 CFU/mL *K. pneumoniae.* When incubation of *K. pneumoniae and P. mirabilis* increases in test 3, conversion of green color of test to yellow was supported by substantial increase in ΔE and G/B values.

**Figure 6:**
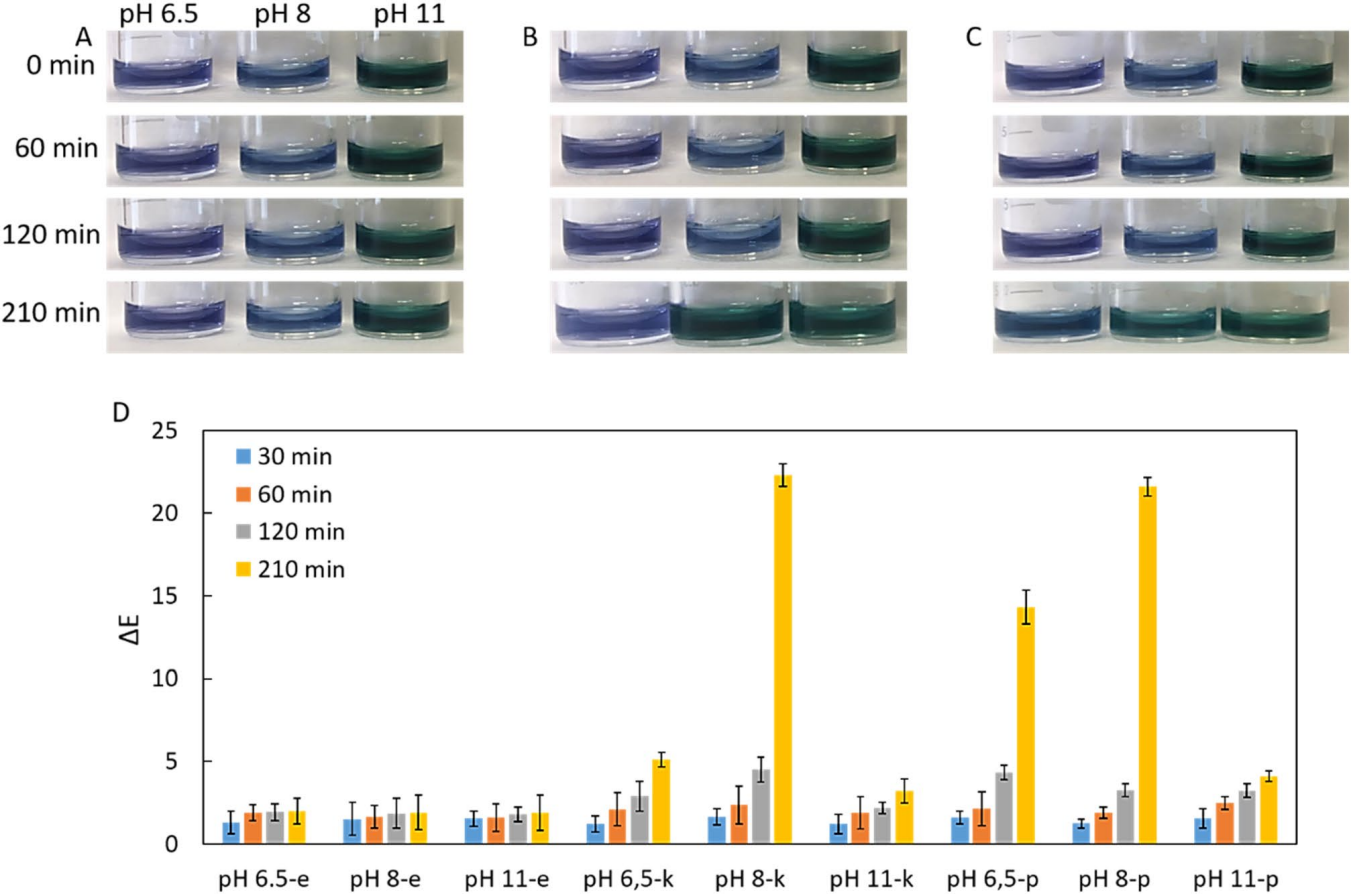
Colorimetric readouts of the urease test with a naked eye for (**A**) *E. coli,* (**B**) *K. pneumoniae* and (**C**) *P. mirabilis*. (**D**) With ΔE analysis. The error bars demonstrate one standard deviation (SD) obtained from three independent measurements (n = 3).

The effect of anthocyanin concentration in three urease tests, such as test 1 (pH 6.5), test 2 (pH 8), and test 3 (pH 11), were investigated in the detection of *E. coli, K. pneumoniae* and *P. mirabilis*. Briefly, 50 µL of each bacterial suspension (1.5 McFarland) was added to 100 µL of each test consisting of 40% w/w RCE:PBS (1:1), then each test was incubated for 210 min. The initial color of each test remained unchanged in the presence of *E. coli* in any incubation time (Fig. 6A). While the *K. pneumoniae* strains only changed to blue color of test 2 to green at the 210. min of incubation as visually detected with a naked eye, *P. mirabilis* strains changed both initial colors of test 1 and test 2 at the same incubation time (Fig. 6B,C). The potential reason to have late colorimetric response in these tests is their much anthocyanin concentration (40% w/w). In other words, there are numerous numbers of anthocyanin molecules in these tests, in order to see clear color change, the certain number of anthocyanin must be deprotonated, which can be achieved with much higher concentration of the bacteria and/or much more incubation time.

The ΔE analysis shows that there were no differences between ΔE values in the presence of *E. coli* (Fig. 6D), as no color change in all tests was observed in Fig. 6A. Interestingly, even if the initial color of test 1 was not visually changed in the detection of *K. pneumoniae*, gradual increases in ΔE values were recorded in 120. min and 210. min of incubation. In addition to that while no clear color change was detected with a naked eye in test 2 at the incubation of 120. min, an acceptable difference in ΔE values was recorded. But a larger difference in ΔE values calculated at the incubation of 210. min in the presence of *K. pneumoniae* is consistent with clear color change. It literally confirms the supportive role of the digital imaging process system. In terms of *P. mirabilis* detection, color differences in test 1 and test 2 were clearly calculated with ΔE values.

**Figure 7:**
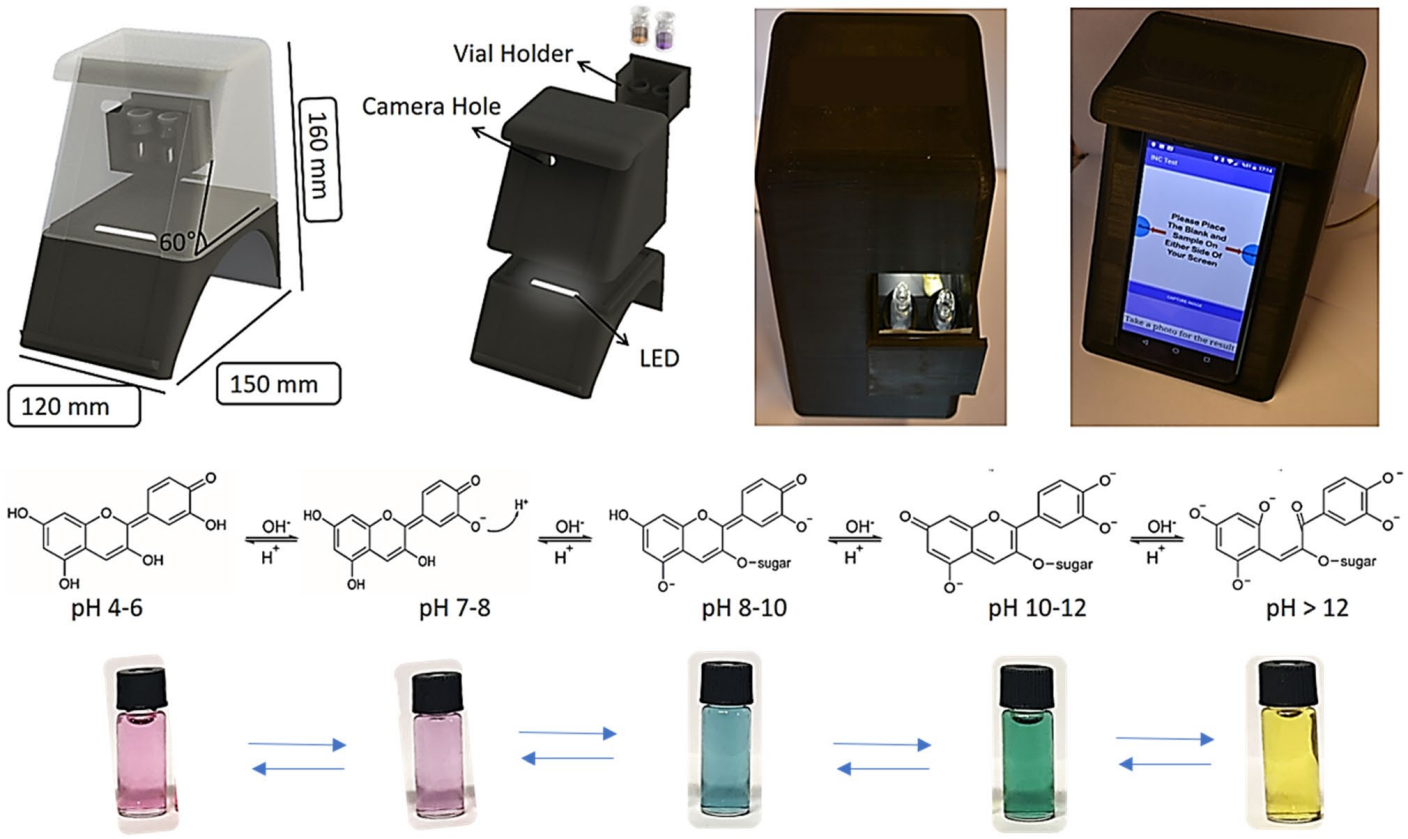
Designing of panel integrated-3D-printed smartphone platform and pH dependent reaction of anthocyanin and color for detection of the pathogens. (SOLIDWORKS Premium 2019 SP4.0 URL link: https://www.solidworks.com/3dexperience-works).

The fabrication of a 3D-printed smartphone platform and the potential reaction of anthocyanin analyzed with digital image processing software (ΔE and RGB) was illustrated in Fig. 7. As detailed in the experimental section, the smartphone platform including the camera, LED, and sample holder was constructed with computer-assisted design (CAD) software. The working mechanism of our tests relies on deprotonation of anthocyanin, which gives various colors based on level of deprotonation or alkaline condition of the reaction environment.

The deprotonation from anthocyanin results in changes in the structure of anthocyanin and electron density, both of which are in charge of changing the color of the urease tests solution. For instance, the molecular structure of anthocyanin in test 1 prepared at pH 6.5 with purple color is the same as given in between pH 4–7 in Fig. 7. Briefly, deprotonation from each hydroxyl group of anthocyanin occurs based upon logarithmic acid dissociation constant (pKa) and the number of deprotonated hydroxyl group increased with the degree of alkaline condition in the urease tests from pH 6.5 to 12. Over pH 12, the aromatic ring on the structure of anthocyanin was hydrolyzed owing to lack of hydroxyl group.

## Conclusions

In conclusion, our study successfully demonstrated that anthocyanin rich-red cabbage (*Brassica oleracea*) extract can be logically prepared as pH-responsive natural indicator and utilized as sensor for detection of model pathogens. We designed anthocyanin based colorimetric urease sensor for portable, swift, sensitive and economic detection of *Proteus mirabilis* and *Klebsiella pneumoniae* by naked eye and 3D-printed smartphone platform. We also provided mechanistic explanation on color change of anthocyanin through deprotonation process against pH changes caused by the bacteria in reaction environment. While growth of *E. coli* was fully inhibited owing to use of the antibiotic and no color change was seen in the tests, presence of *Proteus mirabilis* and *Klebsiella pneumoniae* bacterial strains were colorimetrically detected by a human eye and digital imaging processing (ΔE and G/B analysis). As a part of promising results, the presence of even 1 CFU/mL of *Klebsiella pneumoniae* and 1 CFU/mL of *Proteus mirabilis* were detected in model test. We proposed that the anthocyanin based colorimetric urease sensor with great detection performance and low cost can be implemented in clinics for detection of a wide variety of pathogens in near future.

## Supplementary Information

Supplementary Information 1.

Supplementary Information 2.

## Data Availability

Most of the data generated or analyzed during this study are included in this manuscript and its supplementary information file. Any other raw data are available from the corresponding author on reasonable request.
