# Supplementary material for: Red cabbage extract-mediated colorimetric sensor for swift, sensitive and economic detection of urease-positive bacteria by naked eye and Smartphone platform
Source: Sci Rep. 2023 Feb 4;13:2056. doi: 10.1038/s41598-023-28604-1 (PMC9899230; doi:10.1038/s41598-023-28604-1)

Supplementary Material:

Red cabbage extract-mediated colorimetric sensor for swift, sensitive and economic detection of urease-positive bacteria by naked eye and smartphone platform

Cagla Celik[1,2†], Naim Yagiz Demir[3], Memed Duman[3], Nilay Ildiz[4,*] and Ismail Ocsoy[1,*]

[1]Department of Analytical Chemistry, Faculty of Pharmacy, Erciyes University, 38039, Kayseri, Turkey

[2]Pharmacy Services Program, Vocational School of Health Services, Hitit University, Corum 19000, Turkey

[3]Nanotechnology and Nanomedicine Division, Institute of Science, Hacettepe University, Ankara 06800, Turkey

[4]Department of Pharmaceutical Microbiology, Faculty of Pharmacy, Erciyes University, 38039, Kayseri, Turkey

KEYWORDS:Natural indicator, colorimetric sensor, urease sensor, smartphone application, Proteus mirabilis and Klebsiella pneumoniae

		E.coli (1:4)			
	saline	1 CFU/mL	10 CFU/mL	100 CFU/mL	1000 CFU/mL
10	1,6	1,5	1,3	1,25	1,14
20	1,69	1,5	1,56	1,32	1,25
30	1,74	1,89	1,69	1,4	1,32
45	1,81	2,1	1,74	1,48	1,35
60	1,94	2,15	1,9	1,52	1,79
90	1,99	2,16	2,1	1,84	2,1
120	2,01	3,1	2,3	1,9	2,6
150	2,13	3,18	2,5	2,3	2,8
180	2,2	3,25	2,64	2,5	2,85

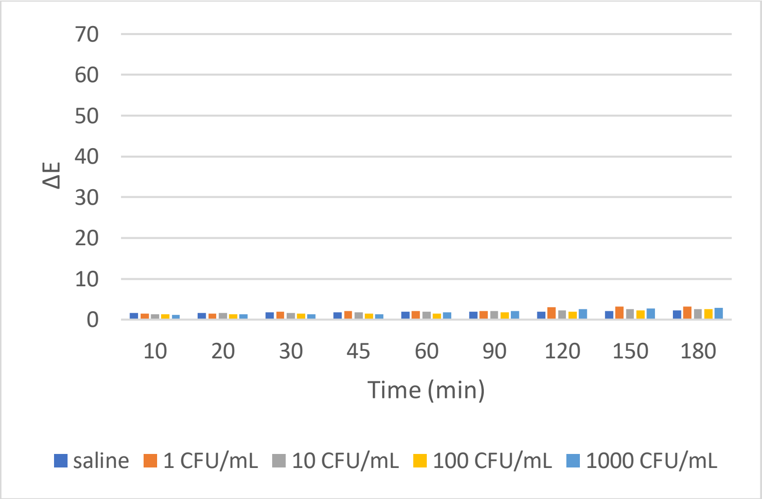

		Klebs (1:4)			
	saline	1 CFU/mL	10 CFU/mL	100 CFU/mL	1000 CFU/mL
10	1,6	1,5	1,3	1,25	1,14
20	1,69	1,5	1,56	1,32	1,25
30	1,74	1,89	1,69	1,4	1,32
45	1,81	2,1	1,74	1,48	1,35
60	1,94	2,15	1,9	1,52	1,79
90	1,99	2,16	2,1	1,84	2,1
120	2,01	3,1	2,3	1,9	2,6
150	2,13	53,5	63,2	64,2	66,9
180	2,2	56,3	65,2	67,4	70,3

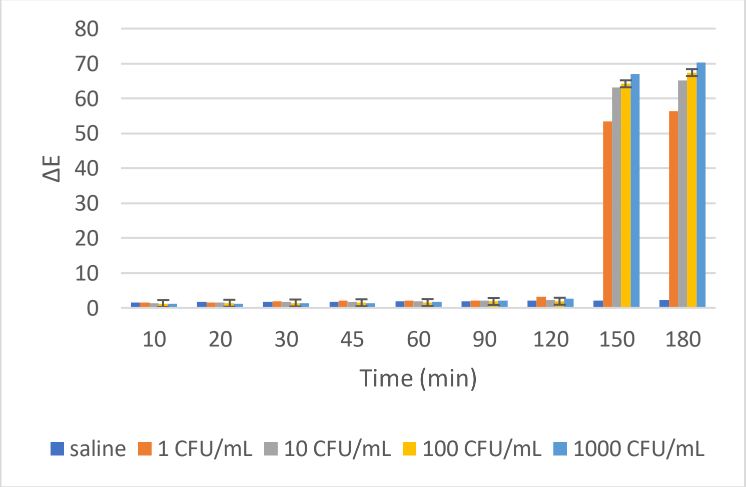

	saline	Proteus (1:4) 1 CFU/mL	10 CFU/mL	100 CFU/mL	1000 CFU/mL
10	1,6	1,5	1,3	1,25	1,14
20	1,69	1,5	1,56	1,32	1,25
30	1,74	1,89	1,69	1,4	1,32
45	1,81	2,1	1,74	1,48	1,35
60	1,94	2,15	1,9	1,52	1,79
90	1,99	2,16	2,1	1,84	2,1
120	2,01	3,1	2,3	1,9	2,6
150	2,13	9,4	10,2	40,02	47,5
180	2,2	36,04	45,01	51,3	64,2

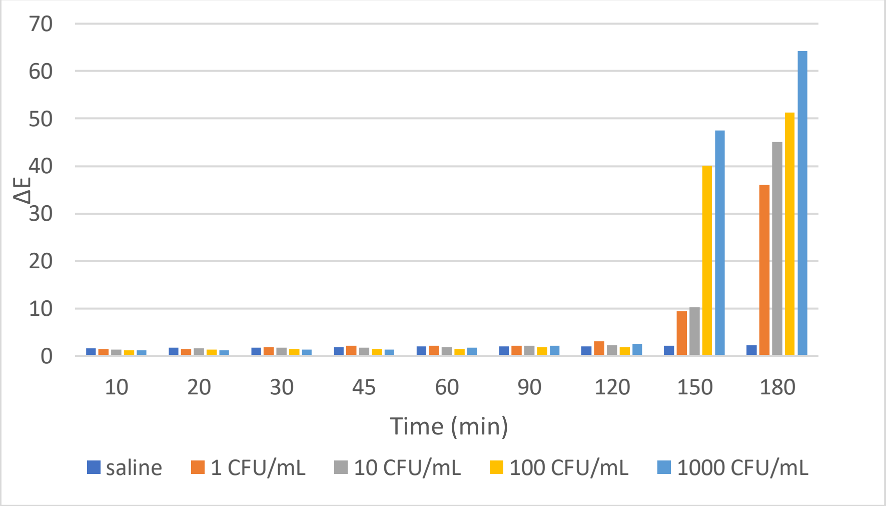

	e.coli (1:4)		
	pH 6,5-e	pH 8-e	pH 11-e
10	1,3	1,5	1,54
20	1,89	1,64	1,6
30	1,92	1,85	1,79
45	1,98	1,9	1,89
60	2,14	2,2	2,12
90	2,5	2,6	2,8
120	2,52	2,5	2,65
150	2,6	2,24	2,71
180	2,69	2,36	2,64

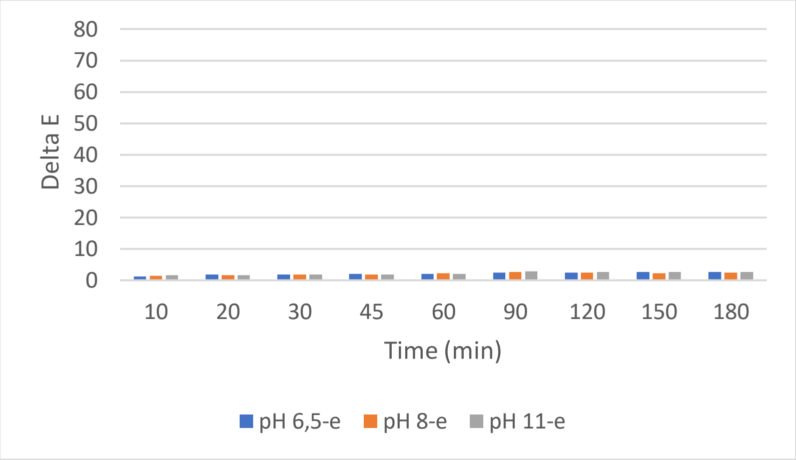

	klebs (1:4)		
	pH 6,5	pH 8	pH 11
10	1,3	1,5	1,54
20	1,89	1,64	1,6
30	1,92	1,85	1,79
45	1,98	1,9	1,89
60	2,14	2,2	2,12
90	2,5	2,6	2,8
120	14,3	10,9	57
150	45,03	51,1	72,8
180	51,3	59,5	74,7

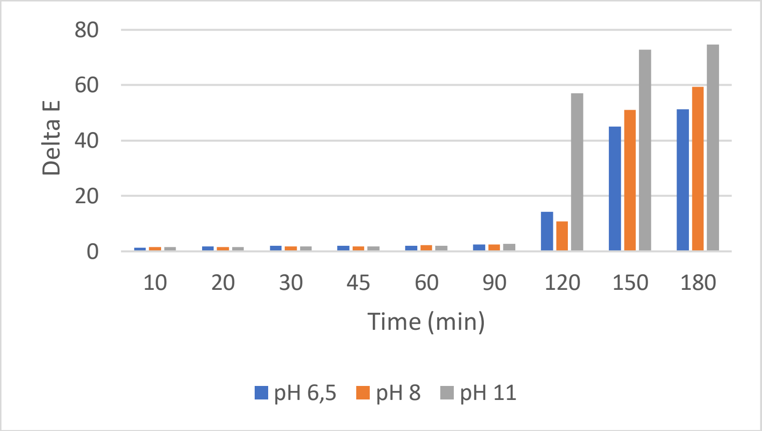

	proteus (1:4)		
	pH 6,5	pH 8	pH 11
10	1,3	1,5	1,54
20	1,89	1,64	1,6
30	1,92	1,85	1,79
45	1,98	1,9	1,89
60	2,14	2,2	2,12
90	2,5	2,6	2,8
120	26,3	17,2	69,3
150	41	33,5	74,07
180	56,4	51,5	80,6

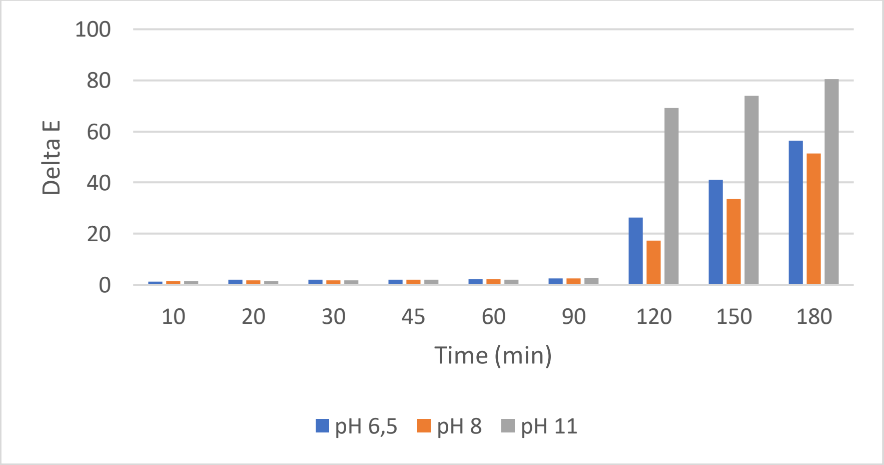

	saline	e. coli (1:1) 1 CFU/mL	10 CFU/mL	100 CFU/mL	1000 CFU/mL
30	1,6	1,89	1,69	1,4	1,32
60	1,69	2,1	1,74	1,48	1,35
120	1,74	2,15	1,9	1,52	1,79
150	1,81	2,16	2,1	1,84	2,1
180	1,94	3,1	2,3	1,9	2,6
210	1,99	3,18	2,5	2,3	2,8

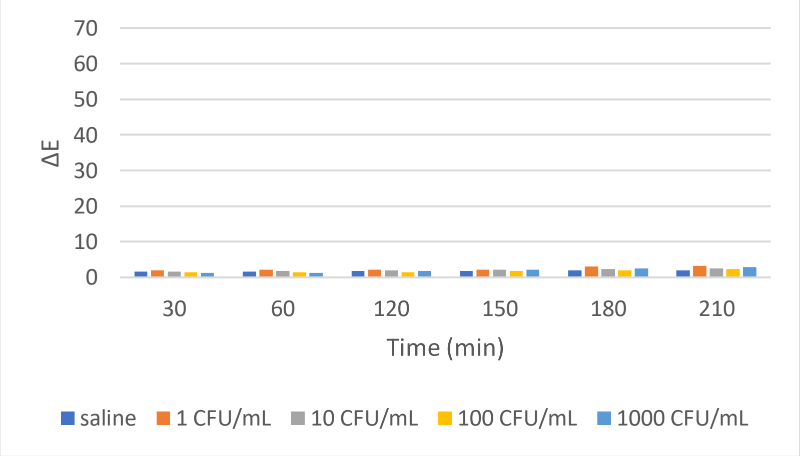

	saline	Klebs (1:1) 1 CFU/mL	10 CFU/mL	100 CFU/mL	1000 CFU/mL
30	1,6	1,5	1,3	2,1	2,13
60	1,69	1,5	1,56	4,5	5,02
120	1,74	1,89	1,69	12,7	14,2
150	1,81	2,1	2,74	16,1	16,9
180	1,94	3,02	13,2	16,2	16,9
210	1,99	9,6	15,1	16,7	17,8

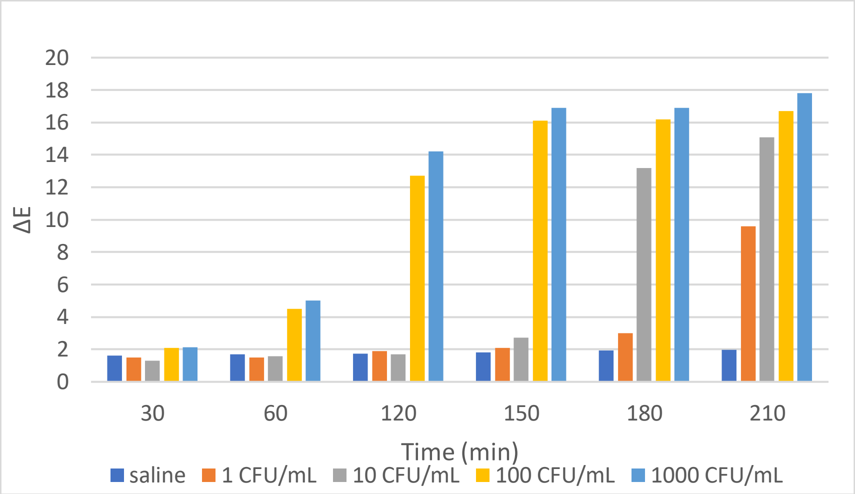

	saline	Proteus (1:1) 1 CFU/mL	10 CFU/mL	100 CFU/mL	1000 CFU/mL
30	1,6	1,5	1,3	1,36	1,2
60	1,69	1,5	1,56	2,1	2,5
120	1,74	1,89	1,69	10,4	11,2
150	1,81	2,1	1,74	10,8	11,3
180	1,94	2,15	1,9	11	12,6
210	1,99	2,16	2,1	11,6	12,9

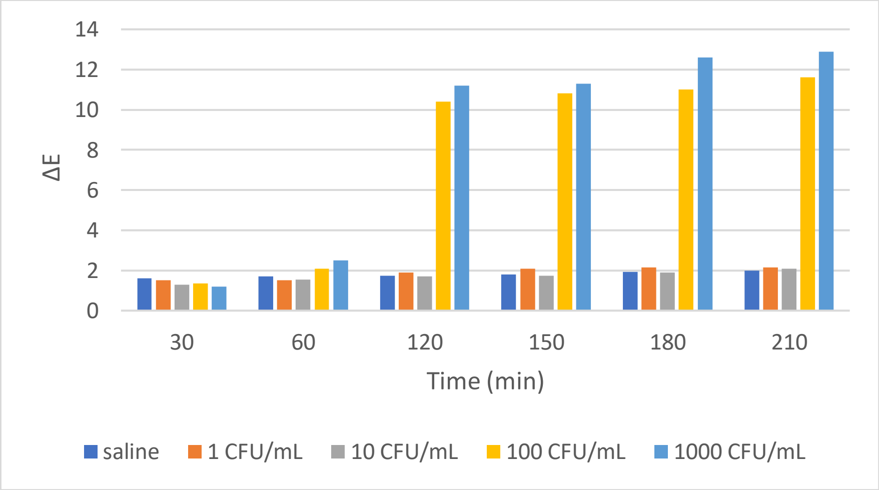

	e.coli (1:1)		
	pH 6,5	pH 8	pH 11
30	1,3	1,5	1,54
60	1,89	1,64	1,6
120	1,92	1,85	1,79
210	1,98	1,9	1,89

	klebs (1:1)		
	pH 6,5-k	pH 8-k	pH 11-k
30	1,2	1,63	1,2
60	2,1	2,36	1,89
120	2,89	4,5	2,18
210	5,1	22,3	3,2

	proteus (1:1)		
	pH 6,5-p	pH 8-p	pH 11-p
30	1,59	1,23	1,56
60	2,14	1,89	2,47
120	4,32	3,25	3,21
210	14,3	21,6	4,1

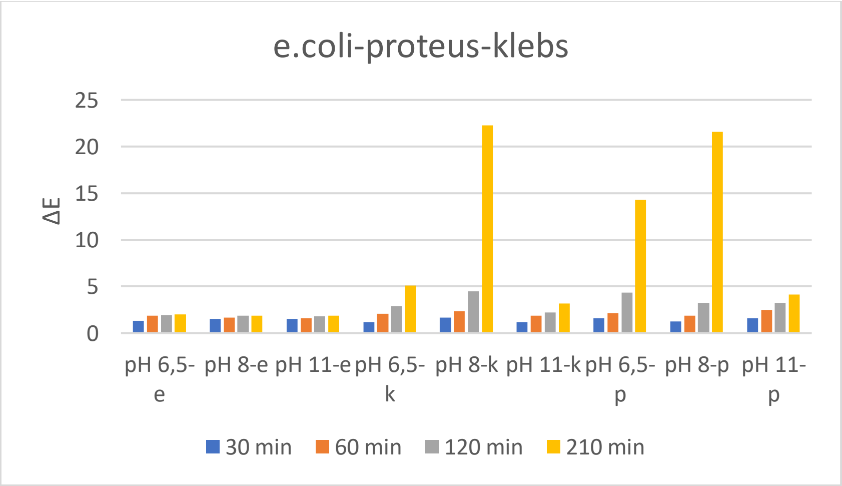

		e.coli (1:4)			
	SF	1	10	100	1000
10	0,75	0,76	0,71	0,75	0,75
20	0,79	0,77	0,72	0,74	0,71
30	0,83	0,754	0,75	0,753	0,74
45	0,79	0,784	0,746	0,76	0,72
60	0,78	0,791	0,736	0,74	0,76
90	0,79	0,788	0,741	0,77	0,74
120	0,78	0,784	0,752	0,72	0,75
150	0,78	0,775	0,744	0,73	0,74
180	0,79	0,72	0,73	0,76	0,75

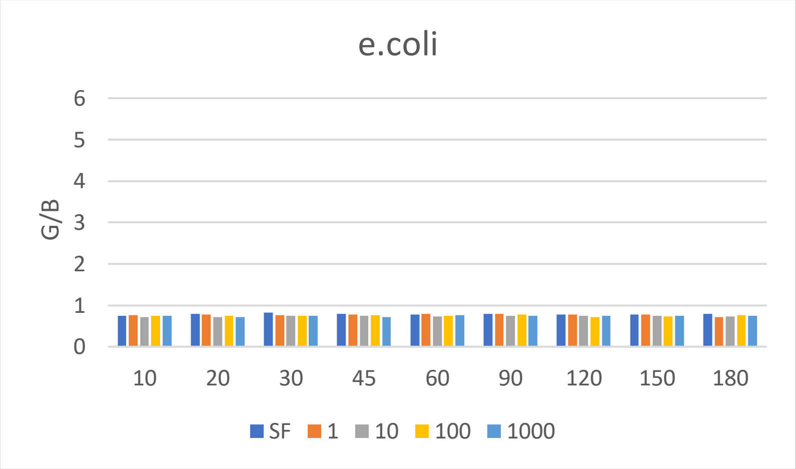

		klebs (1:4)			
	SF	1	10	100	1000
10	0,75	0,76	0,73	0,75	0,74
20	0,79	0,77	0,75	0,746	0,72
30	0,83	0,754	0,74	0,736	0,76
45	0,79	0,784	0,753	0,741	0,74
60	0,78	0,791	0,76	0,752	0,75
90	0,79	0,788	0,74	0,744	0,74
120	0,78	0,74	0,77	0,73	0,75
150	0,78	3,72	4,65	4,88	5,36
180	0,79	3,77	4,72	5,27	5,45

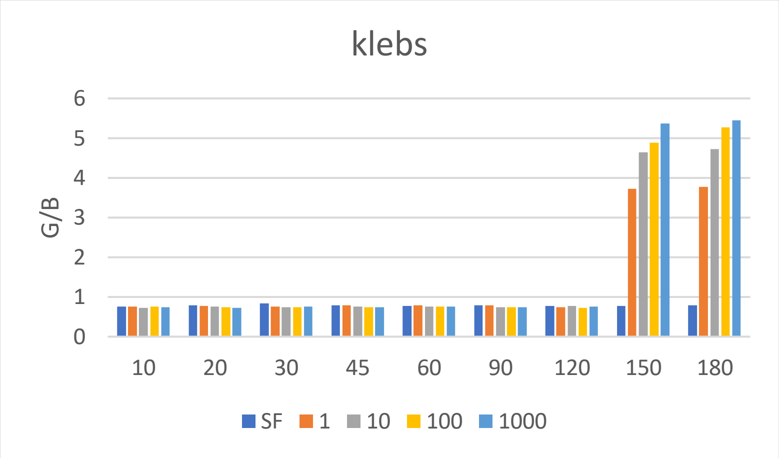

proteus (1:4)					
	SF	1	10	100	1000
10	0,8	0,83	0,75	0,74	0,71
20	0,74	0,79	0,746	0,72	0,72
30	0,73	0,78	0,736	0,76	0,75
45	0,7	0,79	0,741	0,74	0,746
60	0,68	0,78	0,752	0,75	0,736
90	0,71	0,78	0,744	0,74	0,741
120	0,7	0,79	0,73	0,76	0,752
150	0,65	0,84	0,74	2,1	2,7
180	0,62	1,71	2,41	3,14	5,09

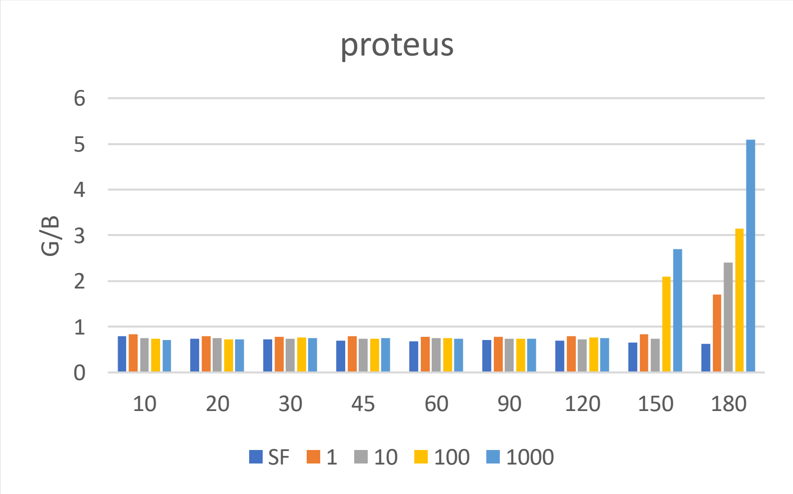

	pH 6,5-e	pH 8-e	pH 11-e	pH 6,5-k	pH 8-k	pH 11-k	pH 6,5-p	pH 8-p	pH 11-p
10 min	0,78	0,71	0,73	0,78	0,71	0,75	0,71	0,65	0,69
20 min	0,75	0,7	0,75	0,74	0,72	0,77	0,72	0,7	0,74
30 min	0,79	0,69	0,74	0,77	0,7	0,78	0,71	0,68	0,71
45 min	0,83	0,67	0,753	0,78	0,75	0,74	0,74	0,69	0,75
60 min	0,79	0,68	0,76	0,79	0,78	0,8	0,71	0,67	0,77
90 min	0,78	0,7	0,74	0,81	0,8	0,86	0,77	0,7	0,87
120 min	0,79	0,69	0,77	0,69	0,87	2,96	0,78	0,94	3,23
150 min	0,78	0,7	0,76	1,82	2,83	5,29	0,84	1,46	5,57
180 min	0,8	0,68	0,75	2,1	3,9	5,33	2,35	3,29	6,76

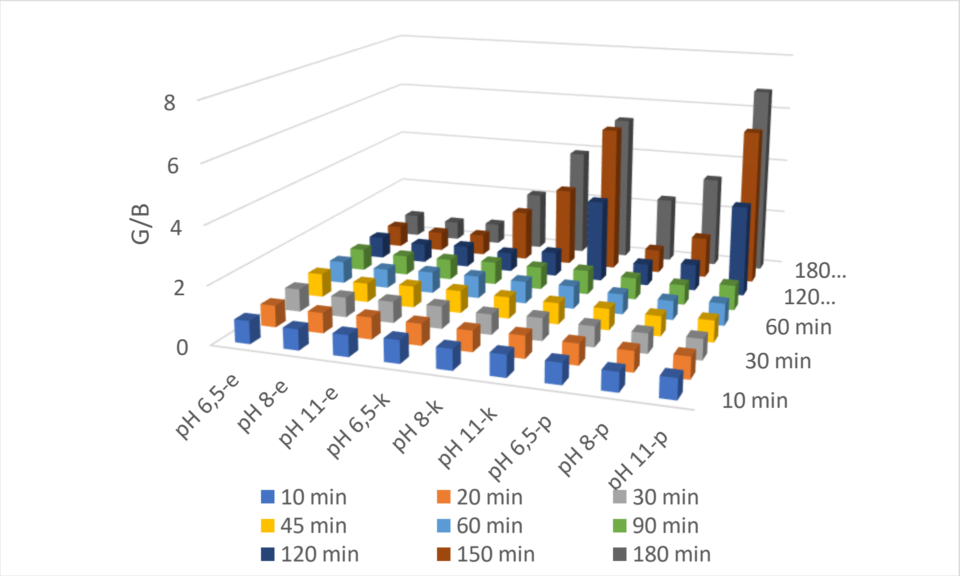

Supplement: Supplementary file 1 — Supplementary Information 1. [file 41598_2023_28604_MOESM1_ESM.pdf]
