# Supplementary material for: Red cabbage extract-mediated colorimetric sensor for swift, sensitive and economic detection of urease-positive bacteria by naked eye and Smartphone platform
Source: Sci Rep. 2023 Feb 4;13:2056. doi: 10.1038/s41598-023-28604-1 (PMC9899230; doi:10.1038/s41598-023-28604-1)
Supplement: Supplementary file 2 — Supplementary Information 2. [file 41598_2023_28604_MOESM2_ESM.docx]

**Supplementary Material:**

Red cabbage extract-mediated colorimetric sensor for swift, sensitive and economic detection of urease-positive bacteria by naked eye and smartphone platform

Cagla Celik^1,2†^, Naim Yagiz Demir^3^, Memed Duman^3^, Nilay Ildiz^4,*^ and Ismail Ocsoy^1,*^

^1^Department of Analytical Chemistry, Faculty of Pharmacy, Erciyes University, 38039, Kayseri, Turkey

^2^Pharmacy Services Program, Vocational School of Health Services, Hitit University, Corum 19000, Turkey

^3^Nanotechnology and Nanomedicine Division, Institute of Science, Hacettepe University, Ankara 06800, Turkey

^4^Department of Pharmaceutical Microbiology, Faculty of Pharmacy, Erciyes University, 38039, Kayseri, Turkey

KEYWORDS:Natural indicator, colorimetric sensor, urease sensor, smartphone application, Proteus mirabilis and Klebsiella pneumoniae


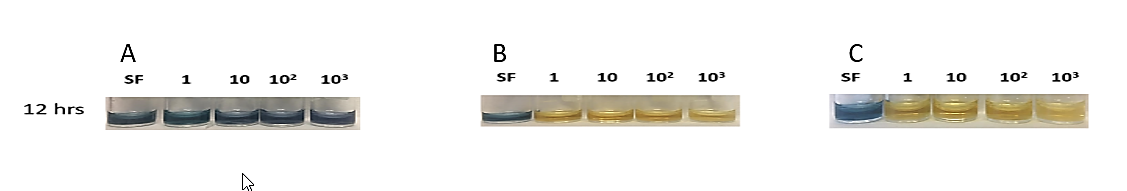


**Figure S1.** Colorimetric readouts of test 2 with a naked eye for A) *E. coli,* B) *K. pneumoniae* and C) *P. mirabilis*.
